# Integrated Pharmacodynamic Analysis Identifies Two Metabolic Adaption Pathways to Metformin in Breast Cancer

**DOI:** 10.1016/j.cmet.2018.08.021

**Published:** 2018-11-06

**Authors:** Simon R. Lord, Wei-Chen Cheng, Dan Liu, Edoardo Gaude, Syed Haider, Tom Metcalf, Neel Patel, Eugene J. Teoh, Fergus Gleeson, Kevin Bradley, Simon Wigfield, Christos Zois, Daniel R. McGowan, Mei-Lin Ah-See, Alastair M. Thompson, Anand Sharma, Luc Bidaut, Michael Pollak, Pankaj G. Roy, Fredrik Karpe, Tim James, Ruth English, Rosie F. Adams, Leticia Campo, Lisa Ayers, Cameron Snell, Ioannis Roxanis, Christian Frezza, John D. Fenwick, Francesca M. Buffa, Adrian L. Harris

**Affiliations:** 1 Department of Oncology, University of Oxford, Churchill Hospital, Oxford OX3 7LE, UK; 2 Molecular Oncology Laboratories, Weatherall Institute of Molecular Medicine, University of Oxford, John Radcliffe Hospital, Oxford OX3 9DS, UK; 3 NIHR Oxford Biomedical Research Centre, Oxford University Hospitals NHS Foundation Trust, Churchill Hospital, Oxford OX3 7LE, UK; 4 MRC Cancer Unit, University of Cambridge, Hutchison/MRC Research Centre, Cambridge Biomedical Campus, Cambridge CB2 0XZ, UK; 5 Breast Cancer Now Research Centre, The Institute of Cancer Research, London SW3 6JB, UK; 6 Institute of Translational Medicine, University of Liverpool, Royal Liverpool University Hospital, Liverpool L69 3GA, UK; 7 Department of Nuclear Medicine, Oxford University Hospitals NHS Foundation Trust, Churchill Hospital, Oxford OX3 7LE, UK; 8 Department of Oncology, Luton and Dunstable Hospital, Luton, UK; 9 Department of Breast Surgical Oncology, MD Anderson Cancer Centre, Houston, TX 77030, USA; 10 College of Science, University of Lincoln, Lincoln LN6 7TS, UK; 11 Clinical Research Imaging Facility, University of Dundee, Ninewells Hospital, Dundee DD2 1SY, UK; 12 Department of Oncology, McGill University, Montreal, QC H3T 1E2, Canada; 13 Breast Surgery Unit, Oxford University Hospitals NHS Foundation Trust, Churchill Hospital, Oxford OX3 7LE, UK; 14 Oxford Centre for Diabetes, Endocrinology and Metabolism, Radcliffe Department of Medicine, University of Oxford, Churchill Hospital, Oxford OX3 7LE, UK; 15 Department of Clinical Biochemistry, Oxford University Hospitals NHS Foundation Trust, John Radcliffe Hospital, Oxford OX3 9DU, UK; 16 Oxford Breast Imaging Centre, Oxford University Hospitals NHS Foundation Trust, Oxford OX3 7LE, UK; 17 Department of Clinical and Laboratory Immunology, Oxford University Hospitals NHS Foundation Trust, Churchill Hospital, Oxford OX3 7LE, UK; 18 Department of Anatomical Pathology, Mater Research Institute, Brisbane 4101, Australia; 19 Department of Cellular Pathology, Oxford University Hospitals NHS Foundation Trust, John Radcliffe Hospital, Oxford OX3 9DU, UK

**Keywords:** metformin, breast neoplasms, positron emission tomography, gene expression profiling, metabolomics, mitochondria, cancer metabolism, clinical study

## Abstract

Late-phase clinical trials investigating metformin as a cancer therapy are underway. However, there remains controversy as to the mode of action of metformin in tumors at clinical doses. We conducted a clinical study integrating measurement of markers of systemic metabolism, dynamic FDG-PET-CT, transcriptomics, and metabolomics at paired time points to profile the bioactivity of metformin in primary breast cancer. We show metformin reduces the levels of mitochondrial metabolites, activates multiple mitochondrial metabolic pathways, and increases 18-FDG flux in tumors. Two tumor groups are identified with distinct metabolic responses, an OXPHOS transcriptional response (OTR) group for which there is an increase in OXPHOS gene transcription and an FDG response group with increased 18-FDG uptake. Increase in proliferation, as measured by a validated proliferation signature, suggested that patients in the OTR group were resistant to metformin treatment. We conclude that mitochondrial response to metformin in primary breast cancer may define anti-tumor effect.

## Highlights

• A pharmacodynamic window study of metformin in patients with breast cancer
• Metformin increases 18-FDG flux in primary breast cancer
• Two distinct metabolic responses to metformin are identified in tumors
• Tumors that upregulate OXPHOS genes show an increase in proliferation score

## Graphical Abstract

**Figure:**
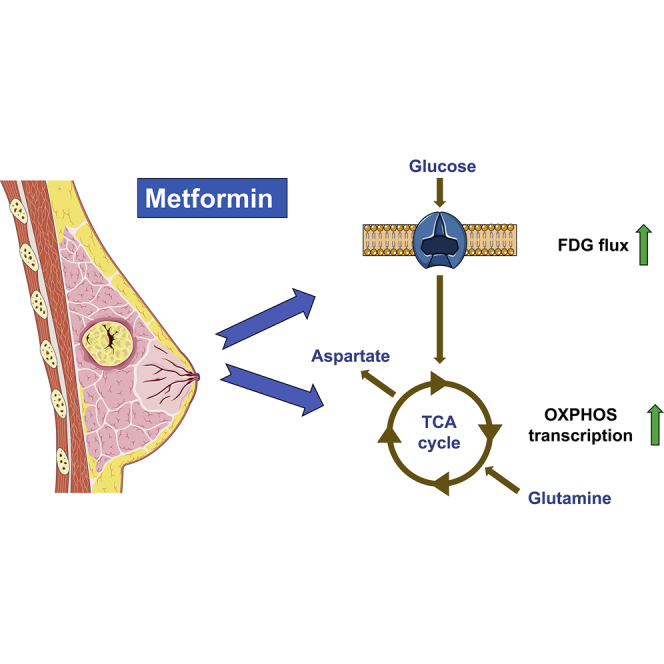

## Teaser

Using dynamic PET imaging, metabolomics, and transcriptomics in breast cancer patients, Lord et al. find that metformin increases 18-FDG flux into tumors and activates mitochondrial metabolism genes. Two distinct metabolic responses are observed and linked to differing proliferation signatures, suggesting that a reactive increase in OXPHOS is linked to metformin resistance.

## Introduction

Metformin can reduce proliferation of cancer cell lines *in vitro* and *in vivo*, and this effect has been ascribed to inhibition of mitochondrial complex 1 (Wheaton et al., 2014). However, the doses of metformin used have typically been 10- to 1,000-fold greater than peak plasma level in humans (Dowling et al., 2012). Hence controversy remains as to whether metformin's effects on tumor metabolism at clinical doses are determined by its direct effects on mitochondria or through its action on systemic metabolism via AMPK-dependent inhibition of hepatic gluconeogenesis and subsequent reduced circulating glucose and insulin levels.

Several window trials have used immunohistochemistry to investigate metformin's clinical effects in breast, endometrial, and prostate cancer. A number have shown that metformin can reduce the proliferation marker Ki67, but no singular mechanism has been clearly demonstrated. Activation of AMPK suggestive of an energy stress has been observed, while other studies have demonstrated reduced pAKT consistent with decreased insulin signaling (Dowling et al., 2015, Hadad et al., 2011, Schuler et al., 2015). Recently published work by Liu et al. comparing the metabolite profile of ten ovarian tumor samples from patients on metformin versus ten control samples (patients not on metformin) demonstrated decreases in the levels of some TCA cycle intermediates and short-chain acyl-carnitines. In addition, the response to metformin seen in the human metabolite profiles could be recapitulated in a mouse model and *in vitro* when nutrient concentrations were limited (Liu et al., 2016). To date, this is the most convincing clinical evidence that metformin has significant and measurable mitochondrial effects at standard therapeutic doses. Here, we present the results of a clinical study that integrates tumor metabolomic profiling with dynamic imaging, transcriptomics, and systemic metabolic markers to further dissect the effects of metformin on systemic and breast tumor metabolism.

**Figure 1:**
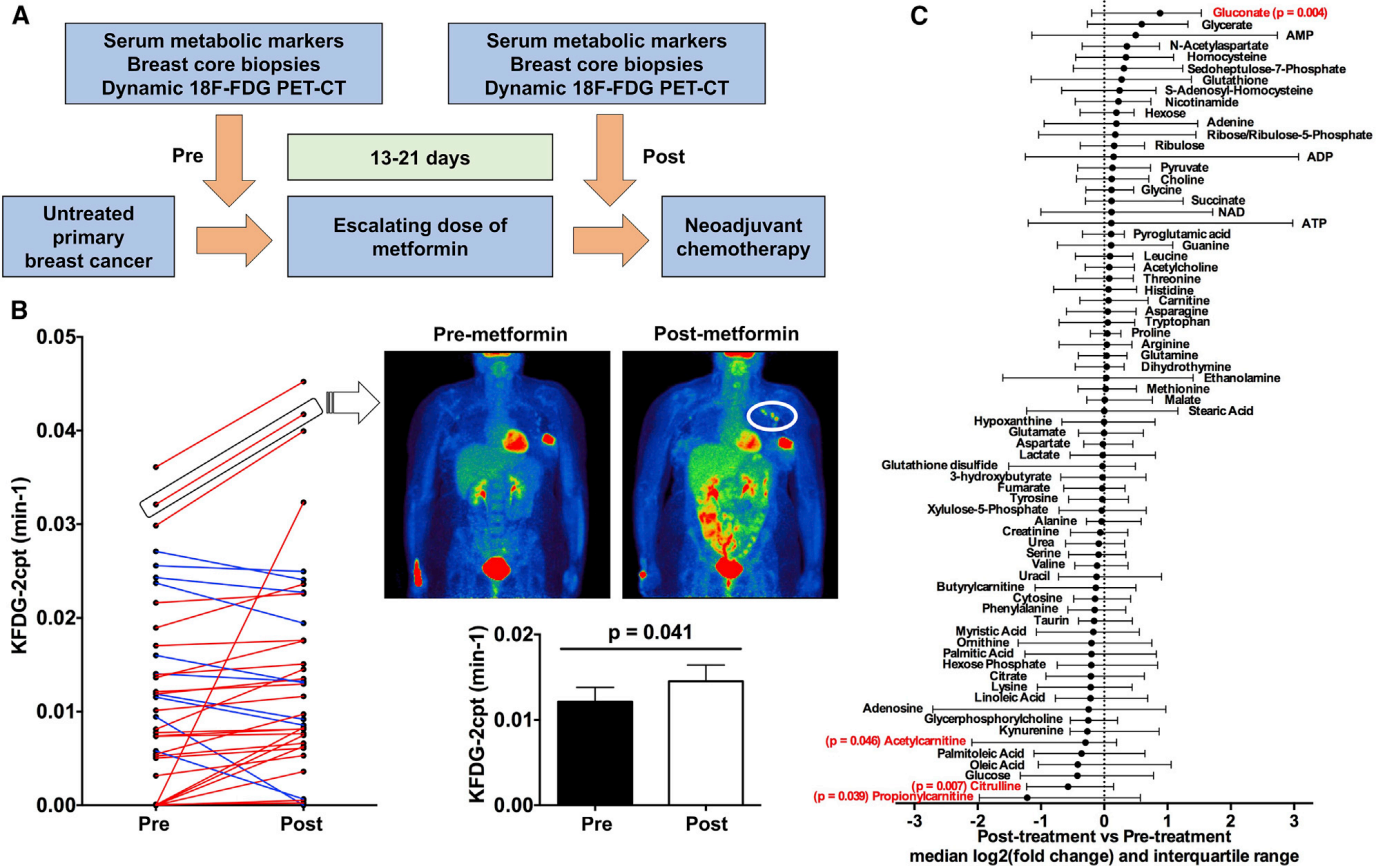
Trial Design and Imaging Analysis (A) Study design. Shortly after diagnosis, patients with untreated primary breast cancer received 13–21 days of slow release metformin at escalating dose levels (500 mg for days 1**–**3, 1,000 mg for days 4–6, and 1,500 mg thereafter) with core biopsies taken pre- and post-metformin before proceeding to neoadjuvant chemotherapy. (B) Change in the FDG flux constant K_FDG-2cpt_ of the primary tumor in individual patients (left panel) and overall (lower right panel) pre- and post-metformin (n = 36, paired t test; data shown are means ± SEM). Upper right panel: static PET-CT images in coronal plane pre- and post-metformin are from an individual with an increase in K_FDG-2cpt_ following metformin; note increased uptake in axillary lymph nodes (circled). (C) Median fold change and interquartile range for metabolites pre- and post-metformin. Metabolites with statistically significant absolute change on Wilcoxon signed rank test are shown in red with p values (n = 29). See also Figure S1 and Tables S1–S3.

We recruited 40 female patients with treatment-naive primary breast cancer to the study. Before and after a 13- to 21-day course of metformin, patients underwent a dynamic fluoro-deoxy-D-glucose positron emission tomography-computed tomography (FDG-PET-CT) scan, breast core biopsies from the primary tumor under ultrasound guidance, and blood samples to assay host metabolic markers of the insulin axis (Figure 1A). See Table S1 for details of study entry criteria and Table S2 for tumor features. See Supplemental Information for further detail.

## Results and Discussion

### Metformin Increases FDG Flux into Primary Breast Tumors

Pre-clinical data have shown that inhibition of oxidative phosphorylation (OXPHOS) by metformin increases dependence on glycolysis (Ben Sahra et al., 2010, Birsoy et al., 2014, Wheaton et al., 2014). The FDG radio-tracer is a marker of tissue glucose utilization. Kinetics analysis of FDG uptake time courses obtained from dynamic PET images potentially provides more consistent measures of tumor tracer uptake, adjusted for variations in tracer inflow to the tumor, than standard static FDG-PET-CT (Dunnwald et al., 2011). Using an irreversible two-tissue compartment model describing rates of FDG transport and phosphorylation (STAR Methods), we observed an increase in FDG flux (K_FDG_) into the primary breast cancer following metformin (Figure 1B) but no change in the static uptake measures SUL_max_ and SUL_mean_ for tumor (standardized uptake values normalized for lean body mass) (Figures S1A and S1B; Table S3). There was no change in nodal SUL_max_ for patients with FDG avidity within ipsilateral axillary lymph nodes (Figure S1C). There was a significant correlation between change in K_FDG_ in the primary tumor and change in SUL_max_ in the axillary nodes (Figure S1D).

The above findings infer that metformin treatment leads to increased glucose uptake by breast tumors and this would be consistent with a switch to glycolytic metabolism. In addition, the analysis emphasizes the sensitivity of dynamic FDG-PET over static scanning in identifying subtle pharmacodynamic changes in glucose metabolism. If normal tissues such as liver absorbed more FDG in response to metformin, FDG activity concentrations in the blood would fall, potentially reducing FDG uptake by the tumor. However, the compartment model/flux constant approach describes tumor FDG uptake after allowing for differences across the whole time course of the dynamic scan in levels of blood-borne tracer flowing into the tumor, determined from imaged activity concentrations in the descending aorta. It is possible that it is precisely because this model controls for the flow of tracer into the tumor that we see a significant change in the flux constant and not standardized uptake values on static PET scanning.

### Two Tumor Groups with Distinct Metabolic Responses to Metformin

We did not observe changes in the levels of the TCA cycle intermediates citrate, succinate, fumarate, and malate in contrast to Liu et al. (2016), or aspartate, a key marker of electron transport chain integrity (Figure 1C). Ornithine is condensed with carbamoyl phosphate to produce citrulline in the only intra-mitochondrial reaction of the urea cycle and citrulline levels decreased (mean log2FC = −0.53; p = 0.007). Some investigators have observed an increase in the ADP/ATP and AMP/ATP ratios typically under *in vitro* nutrient-deprived conditions but there was no significant increase in intratumoral ADP/ATP or AMP/ATP ratios post-metformin (Figure S1E), and this is consistent with metabolomic data from ovarian tumors published in Liu et al. (2016). The discordance in findings with metabolomic profiling from pre-clinical studies may reflect the heterogeneity inherent in a study analyzing clinical samples and the difficulty of making very precise measurements when there may be only small changes in the levels of these metabolites. Mitochondrial dysfunction under the tissue culture conditions described in the literature cited above is likely to be greater than in our study. Indeed, Gaude et al. (2018) showed that, at lower levels of mitochondrial dysfunction, there was little or no decrease in TCA cycle metabolites and aspartate. Uptake from the stroma in an *in vivo* system may help maintain aspartate levels (for example, Birsoy et al., 2015 used a cell line lacking in the transporter SLC1A3, which was expressed at the mRNA level in our clinical samples). In contrast to findings in some other studies (Dowling et al., 2015, Hadad et al., 2011) tumor immunohistochemistry demonstrated no change in AMPK phosphorylation following metformin (paired t test, p = 0.801) (Figure S1F). There was no correlation between change in pAMPK and change in K_FDG_ (Figure S1G).

**Figure 2:**
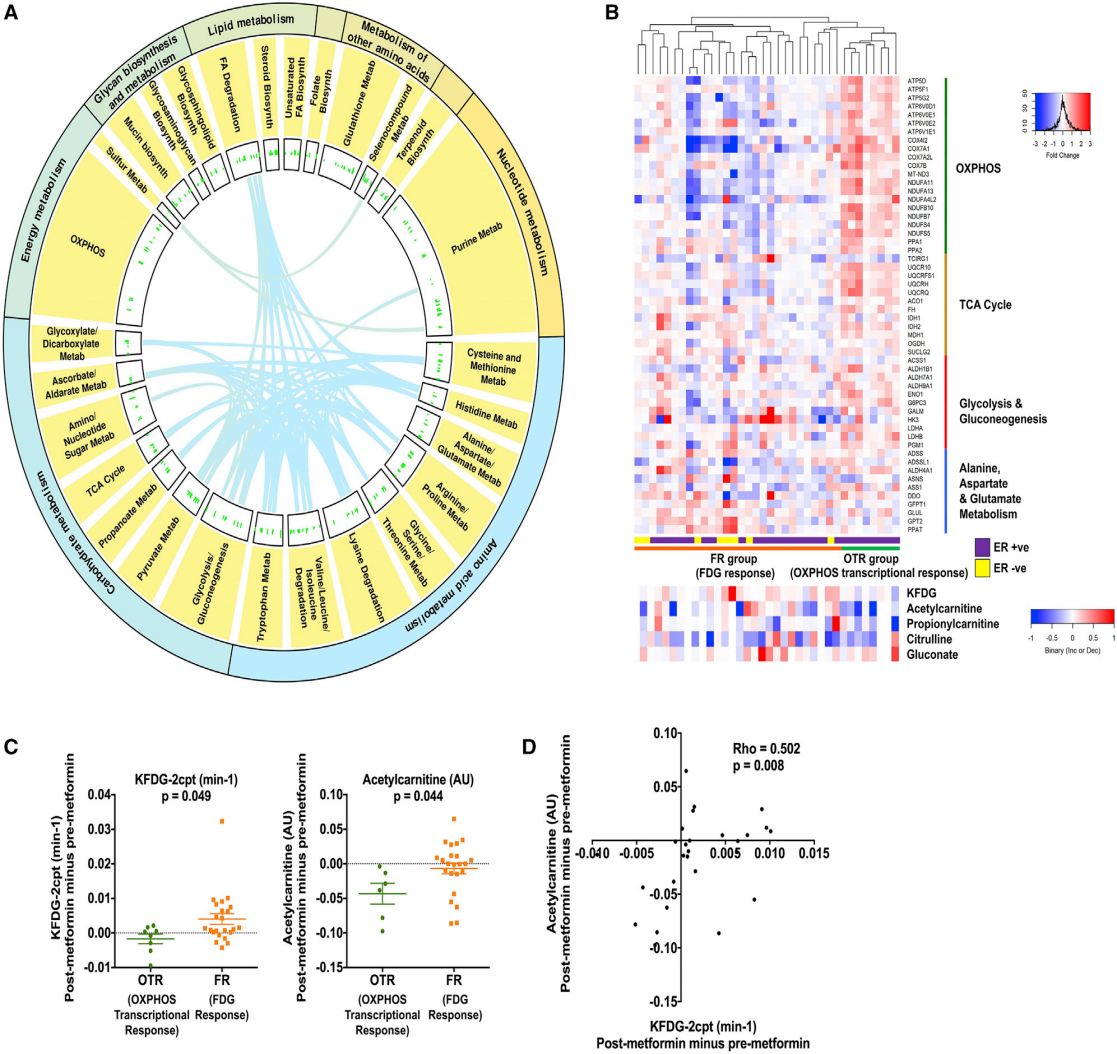
Metformin Alters Levels of Mitochondrial Metabolites and Increases OXPHOS Relevant Gene Transcription in a Subset of Patients (A and B) Circos plot to show all significantly upregulated metabolic pathways in the KEGG database. The width of the outer and inner circles show the mean relative abundances for the broadest hierarchy and secondary hierarchy. The bars in the innermost circle represent the mean relative abundances for genes encoding proteins within the individual pathways. The curved lines link genes that are shared among different pathways as indexed by KEGG (A). Heatmap of differentially expressed genes from the following KEGG pathways: oxidative phosphorylation (KEGG:00190); TCA cycle (KEGG:00020); glycolysis and gluconeogenesis (KEGG:00010); alanine, aspartate, and glutamate metabolism (KEGG:00250). Each row represents a gene and each column represents a single patient (n = 36). Colors reflect the fold change for each gene post-metformin: red, upregulation; blue, downregulation. Samples were visually clustered using hierarchical clustering. OXPHOS transcriptional response (OTR) and FDG response (FR) groups shown. Shown below is heatmap of change in significantly altered metabolites and K_FDG-2cpt_ (all post minus pre) for same individual patients (B). (C) Scatterplot to show for the OTR and FR groups change in K_FDG-2cpt_ and acetylcarnitine levels for the breast primary tumor (both post minus pre). Data shown are means ± SEM, unpaired t test. (D) Correlation between change in K_FDG-2cpt_ and acetylcarnitine (both post minus pre). Spearman's rank correlation coefficient and significance are shown. See also Figure S2 and Table S4.

Whole-transcriptome RNA sequencing pre- and post-metformin revealed significant upregulation of several pathways linked to metabolism (Figure 2A) and more specifically to mitochondrial pathways and disease (Table S4). This included four KEGG pathways that we predicted would be targeted by metformin based on extensive pre-clinical data (Birsoy et al., 2014, Fendt et al., 2013, Liu et al., 2016, Mullen et al., 2011, Wheaton et al., 2014): oxidative phosphorylation (KEGG:00190); TCA cycle (KEGG:00020); glycolysis and gluconeogenesis (KEGG:00010); and alanine, aspartate, and glutamate metabolism (KEGG:00250). Taking all genes that were significantly up- or downregulated from these pathways we observed that for one hierarchical cluster of patients fold change in expression was strikingly increased for this set of genes (OXPHOS responders or OTR [OXPHOS transcriptional response]). All patients in the OTR group were estrogen receptor-positive (Figure 2B). Coherent with this observation, unsupervised hierarchical clustering of the expressed nuclear whole transcriptome showed that patients in the OTR group also clustered together in this analysis (Figure S2A). Notably, clustering of the OTR group also occurred for expressed genes of the mitochondrial transcriptome (Figure S2B). For patients with limited OTR there was evidence of increased glucose uptake defined by an increase in K_FDG_ (FDG responders or FR) in contrast to the OTR group.

**Figure 3:**
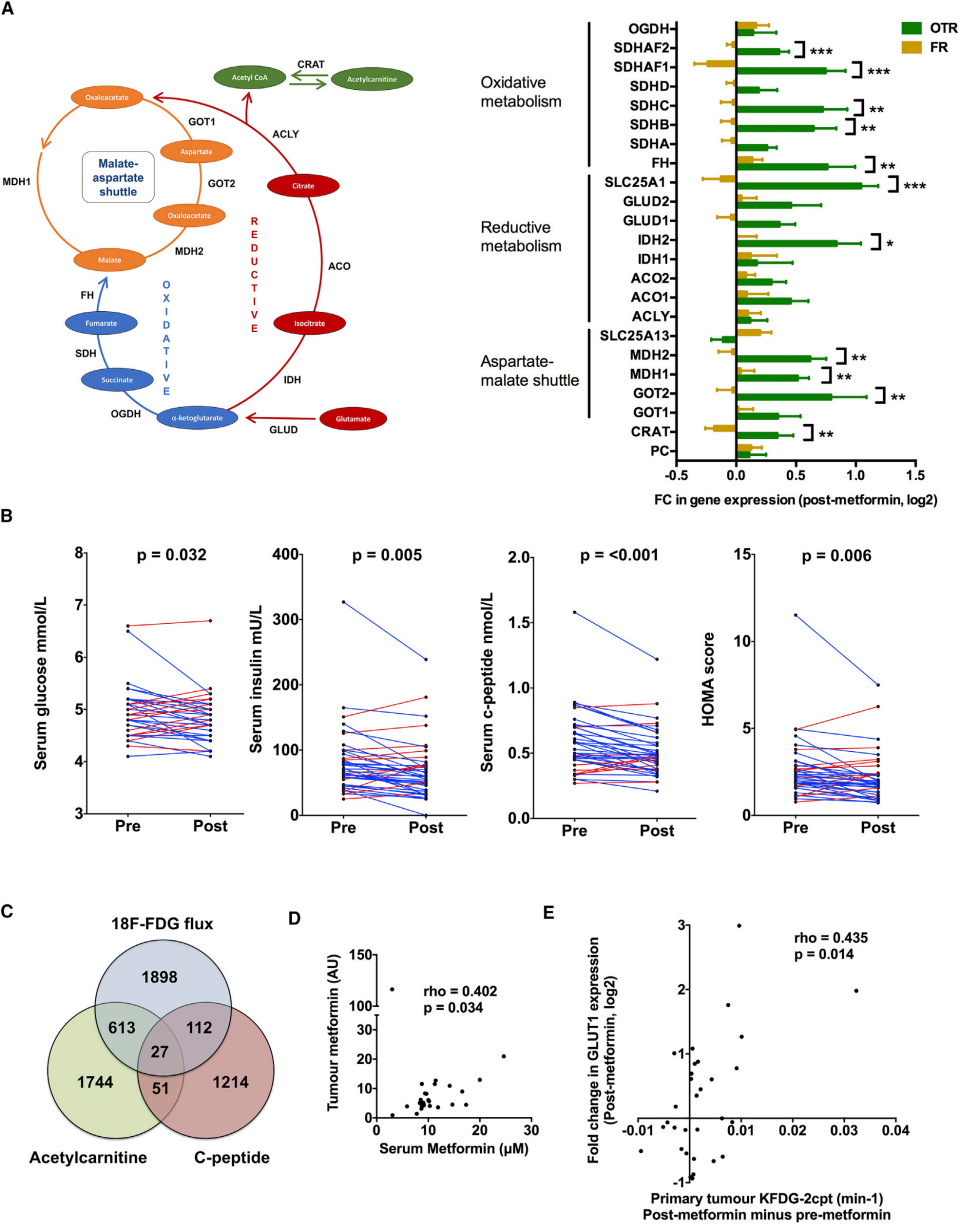
Effect of Metformin on Systemic Metabolism (A) Change in expression of genes involved in regulation of aspartate/malate shuttle and oxidative and reductive metabolism, unpaired t test (n = 36). Data shown are means ± SEM. ^∗^p < 0.05; ^∗∗^p < 0.01; ^∗∗∗^p < 0.001. (B) Pre- and post-metformin serum glucose, insulin, insulin growth factor-1 levels, and HOMA score for individual patients. Significant decrease for each host metabolic marker, p value shown (paired t test, n = 40). (C) Venn diagram to show overlap of all genes whose change in expression correlated with either change in systemic levels of circulating c-peptide or tumor K_FDG-2cpt_ or tumor acetylcarnitine. (D and E) Correlation between peak serum metformin levels (2 hr post dose) and tumor metformin levels (D). Correlation between change in K_FDG-2cpt_ (post minus pre) and GLUT1 expression (log2FC) for the breast primary tumor (E). Spearman's rank correlation coefficient and significance are shown for (D) and (E). See also Figure S3 and Tables S5 and S6.

Consistent with mitochondrial targeting it has recently been shown that metformin treatment leads to a decrease in the levels of short-chain acyl-carnitines in ovarian cancer (Liu et al., 2016). Metabolomic profiling of paired pre- and post-metformin samples showed that acetyl- and propionylcarnitine levels decrease (mean log_2_FC = −1.32, p = 0.046 and log_2_FC = −1.01, p = 0.039, respectively). Acetylcarnitine is a short-chain acyl-carnitine derived from glucose carbons (Schooneman et al., 2013) and, in contrast to the OTR group, their FR counterparts were able to maintain acetylcarnitine levels (Figure 2C). There was a strong correlation between change in K_FDG_ and change in acetylcarnitine levels (Figure 2D). Figure S2C shows the interquartile range and median fold change for metabolites in the OTR and FR groups. It is unclear why intratumoral acetylcarnitine levels dropped, and this finding is at odds with Chen et al. (2016), who showed that complex 1 inhibition in a cell line model resulted in a severalfold increase in acetylcarnitine levels within whole cells and mitochondria. However, this may be due to the discordance between the very different environmental conditions and strength of mitochondrial inhibition in our clinical study compared with cell line models. In addition, Chen et al. only assayed the mitochondrial matrix, and used a different complex 1 inhibitor in a non-breast cancer model. Notably, carnitine o-acetyltransferase, which catalyzes the bidirectional conversion of acetylcarnitine to acetyl-coenzyme A (CoA) within both mitochondria and peroxisomes, was differentially upregulated in the OTR group (all Figure 3A). Hence, we speculate that altered flux in this pathway may be a consequence of metformin treatment. The positive correlation between change in FDG flux and intratumoral acetylcarnitine levels possibly reflects increased flux of glucose carbons toward acetyl-CoA. To fully understand the effects of metformin and mitochondrial defects on acyl-carnitine metabolism will require further work in pre-clinical models.

Maintaining aspartate levels has been shown to be a key resistance mechanism to electron transport chain inhibition and biguanides (Birsoy et al., 2015, Cardaci et al., 2015, Sullivan et al., 2015). There was no difference in aspartate metabolite levels between the FR and OTR groups (Figures 1C and S3A). However, several genes involved in aspartate metabolism were significantly upregulated and it was striking that the increase in expression of three of the five genes that encode for units of the malate-aspartate shuttle (GOT2, MDH1, and MDH2) was significantly greater in the OTR group compared with the FR group (Figure 3A). Dependency on glutamine as a source of citrate for either lipid or aspartate biosynthesis has been shown to be a key resistance mechanism to metformin and other mitochondrial insults (Birsoy et al., 2015, Fendt et al., 2013, Mullen et al., 2011) and we observed increased expression of multiple genes that regulate glutamine metabolism. Two key checkpoints in this process were differentially upregulated in the OTR group, mitochondrial isocitrate dehydrogenase (IDH2) and the citrate transporter, SLC25A1, which delivers glutamine-derived citrate to the cytosol where it is cleaved by ATP citrate lyase to oxaloacetate and acetyl-CoA for aspartate and lipid synthesis, respectively (Figure 3A). Previous work has also shown that both isoforms of isocitrate dehydrogenase, IDH1 and IDH2, support growth in cells that use glutamine-dependent reductive carboxylation. Hence, tumors harboring IDH mutations may be more susceptible to biguanide therapy.

### Systemic Response to Metformin Does Not Correlate with Change in Intratumoral Assays

Metformin has been shown to modulate a number of systemic metabolic and inflammatory markers in diabetic populations. In our study metformin lowered circulating levels of serum glucose, insulin, c-peptide, and an insulin resistance score (homeostatic model assessment or HOMA), but not leptin, adiponectin, C-reactive protein, tumor necrosis factor α, or interleukin-6 (Figures 3B and S3B; Table S5). However, there were no significant differences between the OTR and FR groups in pre-/post-metformin changes in levels of any of these circulating metabolic markers (Figure S3C). There was a marked overlap in genes whose change in expression correlated with change in K_FDG_ and change in acetylcarnitine (hypergeometric test, p < 0.00001), but little corresponding overlap with genes related to change in c-peptide, glucose, insulin, or HOMA (Figures 3C and S3D). Eighteen of the genes correlating with change in K_FDG_ and acetylcarnitine were KEGG-annotated metabolism genes most notably associated with oxidative phosphorylation, carbohydrate, amino acid, and nucleotide metabolism pathways (Table S6). There was an increase in pAKT expression on tumor immunohistochemistry (paired t test, p = 0.026), but no correlation between change in pAKT expression and change in c-peptide, glucose, insulin, or HOMA, and no significant difference between the FR and OTR groups (Figures S3E–S3G). There was also no difference in pAMPK expression between the FR and OTR groups (Figure S3G).

The increase in tumor pAKT expression was unexpected and not consistent with a decrease in insulin receptor signaling or findings in prior studies. AKT activation increases ATP levels in cells and has been identified in a number of studies as being a key player in the regulation of both glycolysis and oxidative phosphorylation (Robey and Hay, 2009). Recent work has shown that mitochondrial AKT activation occurs in the context of tumor energy and hypoxic stress, switching metabolism toward glycolysis (Chae et al., 2016). However, we cannot exclude metformin's systemic effects on host metabolism being a significant factor in modulating tumor metabolism and proliferation, and indeed we would expect a decrease in insulin levels to have some effect on tumor intracellular signaling. Our study only recruited patients with normal systemic glucose levels, and for patients with diabetes or glucose intolerance any effect on insulin signaling via the hypoglycemic activity of metformin is likely to be greater.

We then investigated the relationship between tumor metformin levels and metabolic response. Although serum and tumor levels were significantly correlated with each other (Figure 3D) they did not differ between the OTR and FR groups (Figure S4A). Previously published pre-clinical data suggested that expression of the organic cation transporter, OCT1 (encoded by gene SLC22A1), is required for tumor uptake of metformin and metabolic response (Chandel et al., 2016, Dowling et al., 2016). There was no significant correlation between baseline OCT1 gene expression and tumor metformin levels but notably the patient with highest tumor metformin levels also had the greatest expression of tumor OCT1 (Figure S4B). Furthermore, there was no difference in baseline OCT1 expression between the OTR and FR groups (Figure S4C). Baseline OCT1 expression did correlate with change in K_FDG_, although the relevance of this finding is unclear given that there was no such relationship with tumor metformin levels (Figure S4D).

Glucose transporter gene expression may determine the sensitivity of cell lines to biguanides (Birsoy et al., 2014). Expression of the glucose transporter, GLUT1 (encoded by gene SLC2A1), has previously been shown to correlate with uptake of FDG on PET-CT (Bos et al., 2002), and in our study change in K_FDG_ positively correlated with the change in expression of GLUT1 (Figure 3E). However, there was no significant difference in GLUT1 expression between the two groups although there was for another glucose transporter, GLUT4 (encoded by gene SLC2A4) (Figure S4E).

### OTR to Metformin Relates to Change in a Proliferation Metagene

**Figure 4:**
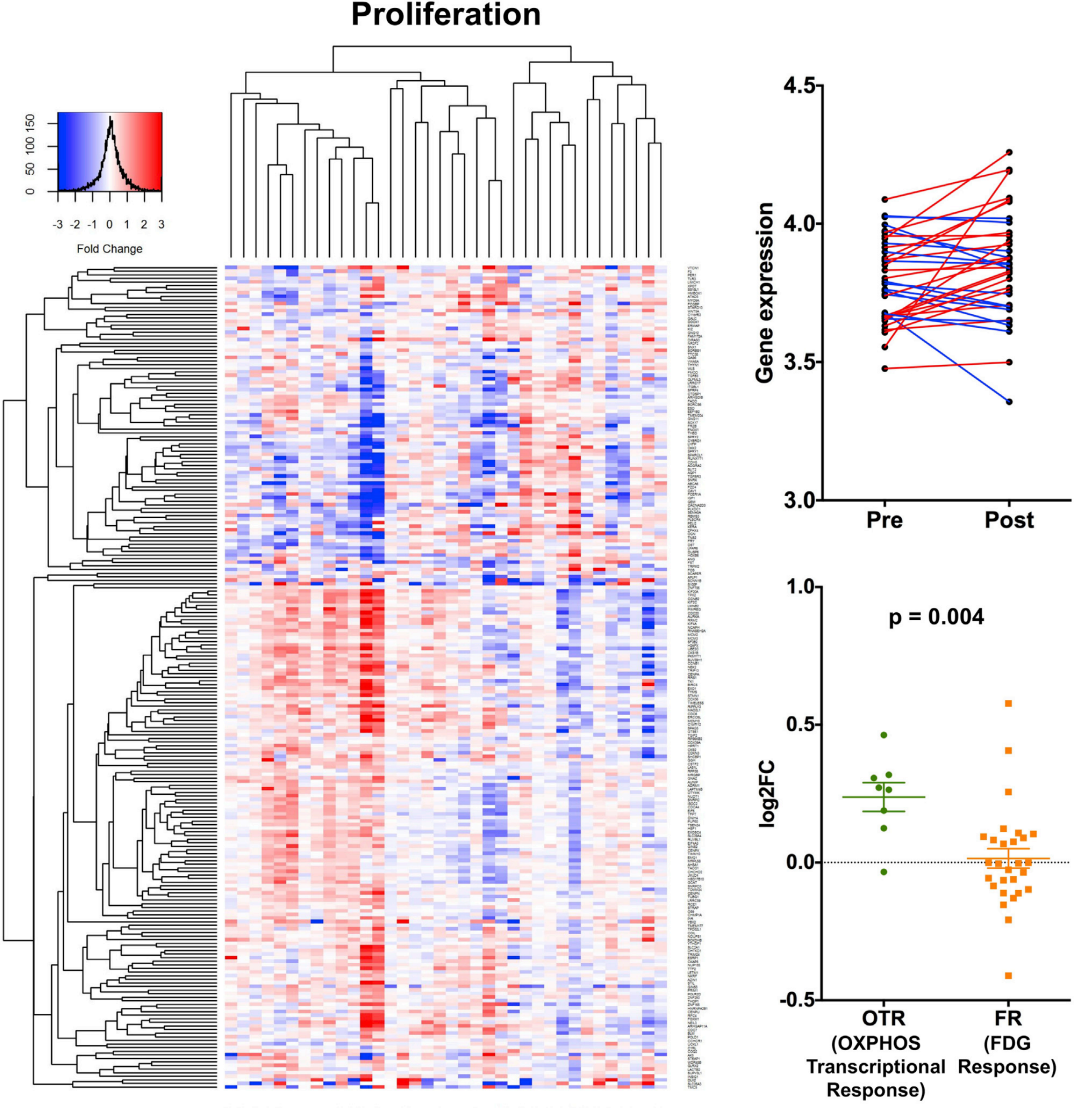
Effect of Metformin on Proliferation Left panel: heatmap of genes from the proliferation signature. Each row represents a gene and each column represents a single patient. Colors reflect the fold change for each gene post-metformin: red, upregulation; blue, downregulation. Samples were visually clustered using hierarchical clustering. Right upper panel: pre- and post-metformin expression of signatures for individual patients (n = 36); right lower panel, scatterplot to show change in expression of signatures for the OTR and FR groups. Data shown are means ± SEM, unpaired t test (n = 36).

Several clinical studies have shown that metformin can reduce breast, prostate, and endometrial cancer cell proliferation (Hadad et al., 2011, Joshua et al., 2014, Laskov et al., 2014, Mitsuhashi et al., 2014, Niraula et al., 2012, Schuler et al., 2015). We explored the effect of metformin on a validated human breast cancer proliferation signature (Desmedt et al., 2008) and overall observed no significant change following metformin treatment (Figure 4). However, it was striking that an increase in metagene expression occurred in the OTR group, while, in contrast, there was a decrease for several patients in the FR group, the change in metagene expression consequently differing significantly between the two groups (Figure 4). Under *in vitro* low-glucose conditions the ability for cell lines to upregulate OXPHOS predicts for sensitivity to biguanides (Birsoy et al., 2014), and our data suggest that a reactive increase in OXPHOS and aspartate synthesis gene transcription may be critical for resistance to metformin. None of the circulating or tumor immunohistochemical markers, metformin levels, K_FDG_, or significantly altered metabolites correlated with change in expression of the proliferation metagene (Figure S4F).

### Conclusion and Perspectives

Our work outlines two types of breast cancer metabolic response to metformin and links the effects of metformin on mitochondrial metabolism with its effects on breast cancer proliferation at a transcriptional level. Tumors that were able to upregulate OXPHOS gene transcription in response to metformin showed an increase in their proliferation score suggestive of resistance following metformin treatment.

The upregulation of multiple transcriptomic pathways involved in mitochondrial metabolism and decrease in levels of several intratumoral mitochondrial metabolites is suggestive of metformin interfering with mitochondrial metabolism. Furthermore, the increased expression of multiple genes regulating glycolysis and glucose transport alongside our imaging data is consistent with mobilization of glucose metabolism in response to metformin. The upregulation of key regulatory genes for glycolysis, aspartate, and glutamine metabolism in response to metformin may represent a mechanism of resistance and confirms the potential of previously proposed strategies to target these pathways, for example, by combining biguanides with glutaminase inhibitors or dichloroacetate (Fendt et al., 2013, Haugrud et al., 2014). Estrogen receptor expression may also act as a biomarker to distinguish the two types of metabolic response. Among the most likely determinants for resistance in our view are mitochondrial defects (for example, mutations in complex 1 genes), and this would be consistent with *in vitro* data (Birsoy et al., 2014). Hence, we propose that translational work within ongoing phase 3 trials should investigate whether mitochondrial mutations herald biguanide sensitivity and clinical outcome. However, we emphasize that early dynamic monitoring of response may detect the heterogeneity that cannot be detectable at baseline.

There have been a number of other window studies designed to assess metformin's pharmacodynamic effects in several different tumor types and it is important to note the differences to this study. Most of these trials have used immunohistochemical approaches on a wide range of markers, but in particular Ki67, AMPK, and markers of apoptosis with discrepant results (Dowling et al., 2015, Hadad et al., 2011, Schuler et al., 2015). The most in-depth clinical study to date to use metabolomic approaches, Liu et al. (2016), suggested some evidence of mitochondrial interference but, in contrast to our study, did not take serial biopsies to allow identification of differential types of response and was limited to effectively one assay. In addition, Liu et al. assayed samples taken from ten patients with ovarian cancer who happened to be receiving metformin for diabetes while using control samples from non-diabetic patients with a lower mean body mass index. Hence, this was a comparison between two patient groups with distinct host metabolism (Liu et al., 2016). In contrast, this study only recruited from a non-diabetic population, the focus of ongoing phase 3 trials.

These data are consistent with several of the observations seen previously using *in vitro* and *in vivo* models but it is still uncertain whether these perturbations are enough for metformin to deliver clinical benefit to patients. A recent substudy of the ALTTO phase 3 adjuvant breast cancer trial reported a strong association between metformin and improved overall survival in diabetic patients (Sonnenblick et al., 2017). Our observations make the case for the continued clinical study of metformin and more potent biguanides (Zhang et al., 2016) in non-diabetic patients. The results of ongoing phase 3 trials are awaited (Gillessen et al., 2016, Goodwin et al., 2015).

### Limitations of Study

This study had no control arm and hence it is possible that some of the observations could be related to the passage of time or interventions (e.g., biopsies). Given the nature of this clinical translational study the analysis especially relies on correlative evaluation and hence we cannot rule out a link between the systemic effects of metformin and significant changes in tumor metabolism and proliferation. Although 40 patients are a substantial number for an involved pharmacodynamic study of this type, correlations across the assays were not able to be carried out across the full cohort for varied reasons (technical difficulties with some scans and insufficient sample to carry out all assays, etc.) and increased recruitment would provide greater power for the analyses.

## STAR★Methods

### Key Resources Table

**Table:** 

REAGENT or RESOURCE	SOURCE	IDENTIFIER
**Antibodies**		

pAMPK thr172 residue	Cell Signaling Technologies	RRID: AB_331250
pAKT ser473 residue	Cell Signaling Technologies	RRID: AB_2315049

**Critical Commercial Assays**		

IL-6	Invitrogen	BMS213HS
TNF-alpha	Invitrogen	BMS223HS
NEBNext mRNA Library Prep Master Mix Set	New England Biolabs	E6110

**Deposited Data**		

Analysed RNASeq data	This paper	http://dx.doi.org/10.17632/cytrpb62f2.1

**Software and Algorithms**		

RNASeq analysis	R-project	v3.3.1
Imaging analysis	Matlab	N/A
Other statistical analyses	GraphPad PRISM	v6.0

**Other**		

Metformin	Bristol-Myers Squibb	Glucophage XR
ClinicalTrials.gov Identifier	N/A	NCT01266486

### Contact for Reagent and Resource Sharing

Further information and requests for resources should be directed to and will be fulfilled by the Lead Contact, Simon Lord (simon.lord@oncology.ox.ac.uk).

### Method Details

#### Clinical Study Design and Patient Selection

Patients were recruited from the medical oncology breast cancer clinic over a period of 30 months between May 2011 and November 2013 in three UK centres, Oxford, Luton and Dundee. Informed consent was obtained from all patients. The study was prospectively approved by the NHS Oxfordshire Research Ethics Committee A and registered with the ClinicalTrials.gov identifier: NCT01266486. All patients at the point of recruitment had been referred with a view to neoadjuvant chemotherapy, had histologically confirmed breast cancer and gave informed consent. In all cases the primary breast cancer was *in situ* and no patients had received any prior treatment for breast cancer. See Table S1 for a list of key eligibility criteria. In total 41 patients were recruited and had evaluable data. See Table S2 for numbers of patients with sufficient data for paired analysis for each assay. All patients were female and the median age at study entry was 49 years (range 27 – 67 years). Median body mass index at study entry was 28.1 (range 19.6 – 45.3).

Metformin was given in the Glucophage XR formulation in an escalating dose once daily for a minimum of 13 days and a maximum of 21 days (500mg for days 1**–**3, 1000mg for days 4**–**6 and 1500mg thereafter). The day prior to commencing metformin a core biopsy was taken under ultrasound guidance from the periphery of the primary tumour. Within 1 minute of this procedure the biopsy material was snap frozen in liquid nitrogen prior to storage at -80°C. Prior to metabolomics analysis biopsy samples were divided and one portion used for broad metabolomics analysis and the other to generate a lipid profile.

#### PET-CT Protocol

The radiotracer, 2-deoxy-2-(18F)fluoro-D-glucose (^18^[F]-FDG), was used for all examinations. Prior to scanning, patients were fasted overnight for at least 8 hours but could drink water. Patients’ blood glucose was checked just prior to the scan with a portable blood glucose monitor to ensure it was <7mmol/L. All scans took place on either a 3D mode time of flight GE Discovery 690 64-slice PET-CT system (GE Healthcare, Milwaukee) or Siemens Biograph mCT-128 (Siemens Healthcare, Germany).

A dynamic acquisition of the breast tumour (and any lymph nodes within the PET field of view) was initiated with the patient imaged supine. Patients were injected with ^18^[F]-FDG (3 MBq/kg, up to a maximum of 400 MBq) 30 seconds into PET imaging, which continued for 45 minutes. The 45 minutes of data were then reconstructed as a sequence of images describing average activity concentrations during a series of time frames (1x30s, 12x5s, 6x10s, 5x30s, 10x60s, 6x300s).

50 minutes after injection, a static PET scan was performed from skull base to mid-thigh, acquiring data for four minutes at each bed position. Thus the primary breast cancer was scanned at approximately 60 minutes post injection, in addition to the dynamic PET scanning from 0-45 minutes. Prior to each PET acquisition a CT scan was performed for localization and PET attenuation correction, using a pitch of 0.984, 120 kV, automA with a noise index of 25.

The PET images were reconstructed on a matrix of 5.5×5.5×3.3 mm^3^ voxels using filtered back projection for the dynamic sequence, and iterative reconstruction for the static scan. See Supplemental Information for further details of static and kinetic analysis of imaging.

#### Static PET-CT Analysis

Tumour volumes were delineated on the 60-minute static FDG-PET scans by a nuclear medicine radiologist working on a Hermes workstation and using Hybrid viewer software (Hermes Medical Solutions AB, Stockholm, Sweden). Maximum and mean standardized uptake values (SUV) within each tumour volume were normalized by lean body mass (LBM) and reported as SUL_mean_=SUV_mean_^∗^LBM/BW and SUL_max_=SUV_max_^∗^LBM/BW respectively, where

$${\text{LBM}}_{\text{Hume}}=\{\begin{array}{c} \text{0}\text{.}32810\;\text{×}\;\text{BW}\;+\;\text{0}\text{.}33929\;\text{×}\;\text{H}\;\;\text{×}\;29\text{.}5336\;\left(\text{Males}\right)\\ \text{0}\text{.}29569\;\text{×}\;\text{BW}\;+\;\text{0}\text{.}41813\;\text{×}\;\text{H×}\;43\text{.}2933\;\left(\text{Females}\right) \end{array}$$

and BW and H are the body weight in kg and height in cm (Hume, 1966).

#### Dynamic PET-CT Analysis

The tumour volume contoured on a static PET-CT image was transferred to the corresponding 0-45 minute dynamic FDG-PET scan by co-registering the two image sets. Time-activity curves (TACs) describing time-courses of mean tumour FDG activity concentration within the tumour were then calculated for the tumour regions of the dynamic scans. Time-courses of blood-borne tracer concentrations were similarly obtained from regions defined in the descending aorta (average 42 ± 4 slices with mean volume of 32 ± 11 cm^3^), and used to describe tracer inflow into tumours (‘input functions’, IF).

Kinetic analysis of tumour FDG uptake was carried out for 36/40 patients, using irreversible 2- and 3-tissue compartment models (Bertoldo et al., 2001). Tumour TACS for the remaining four patients were not analysed as they showed pronounced discontinuities, likely due to movement during scanning. The compartment models characterize FDG transport and intracellular phosphorylation using a small number of parameters, and enable modelled tumour TACs to be calculated directly from IFs. The models were fitted by adjusting the parameters to achieve the best weighted least-squares match between modelled and measured tumour TACs (Liu et al., 2014).

The 2-tissue compartment model (2cpt) provided better descriptions of tumour TAC data, judged by the Akaike and Bayesian information criteria (AIC and BIC) used alongside a runs-test. From each fit, estimated values and associated statistical uncertainties were obtained for the model parameters v_B_, K_1_, k_2_ and k_3_, which respectively describe the fractional tumour blood volume and rate-constants for FDG transport back and forth between the vasculature and tumour cells, and for intra-cellular phosphorylation. Uncertainties on these fitted parameters are quite large due to statistical noise in dynamic PET images. Flux constants K_FDG_, numerically equal to K_1_k_2_/(k_2_+k_3_), were also calculated. Conceptually K_FDG_ describes the rate of intra-cellular FDG phosphorylation when a steady-state unit concentration of FDG exists in the blood, and statistically it is estimated substantially more precisely than the individual model rate-constants. Figure S5 summarizes the analysis. Significances of differences in model parameters before and after metformin were assessed using paired t-test and Wilcoxon signed rank test. Only changes in K_FDG_ proved significant.

It was not useful to kinetically analyse FDG uptake time-courses in the axillary nodes, since the small nodal volumes led to a high degree of noise on the time-courses and fitted kinetics parameters including the flux constant. All patients included in the axillary node analysis who had lymph node avidity had evidence at pre-treatment biopsy or surgery of metastatic breast carcinoma involvement within the axillary nodes with the exception of 3 patients for whom no biopsy or surgical data was available.

#### Mass Spectrometry Analysis of Clinical Samples for Metabolomic Profile

Breast cancer tissue was pulverised via mechanical disruption (IKA Ultra-Turrax T-8 homogenizor) prior to hydrophilic extraction of intracellular metabolites from tissue using a methanol/acetonitrile/water (50/30/20) extraction solution (250 μL of extraction solution per 10mg homogenised tissue). Following thorough mixing, the samples were centrifuged for 10 minutes at 10,000G and the supernatant stored at -80°C prior to mass spectrometry analysis.

For the LC separation, column A the sequant Zic-pHilic (150 mm × 2.1 mm i.d. 5 μm) with the guard column (20 mm × 2.1 mm i.d. 5 μm) from HiChrom, Reading, UK. Mobile phase A: 20 mM ammonium carbonate plus 0.1% ammonia hydroxide in water. Mobile phase B: acetonitrile. The flow rate was kept at 180 μL/minute and gradient as follow: 0–1 minutes 70% of B, 16 minutes 38% of B, 16.5 minutes 70% of B, 25 minutes 70% of B. The mass spectrometer (Thermo Q-Exactive Orbitrap) was operated in a polarity switching mode. Experimenters analysing samples from metabolomics experiments were blinded to the experimental interventions. Samples were randomised in order to avoid machine drifts.

#### Analysis of RNASeq Data

Next generation sequencing of ‘Poly (A) targeted’ mRNA, including library preparation, was carried out by the Oxford Genomics Centre core facility at the Welcome Trust Centre for Human Genetics. The NEBNext mRNA Library Prep Master Mix Set (New England Biolabs) was used for preparation of the expression libraries and the Illumina HiSeq 2000 system used to carry out the sequencing.

Paired-read were aligned to human reference genome GRCh38, including transcriptomic information, by Bowtie 2.2.6 and Tophat v2.1. The fold change of normalized expression level, FPKM (Fragments Per Kilobase of transcript per Million mapped reads), for each gene was then estimated from those aligned reads using Cuffdiff 2.2.1. Non-parametric rank product (R package and version) was used to discover the genes with consistent statistically significant fold change (probability of false positive < 0.05) between pre- and post-metformin treatment, among all patients were selected. This approach was preferred with respect to EdgeR (Anders and Huber, 2010) and Deseq (McCarthy et al., 2012, Robinson et al., 2010) as in datasets with high variability and paired samples (pre and post- treatment) non parametric methods tend to work better in our previous studies (Mehta et al., 2016); however analysis with EdgeR (version 3.16.5) and Deseq (version 1.26.0) was also done and did not change the main conclusions.

#### Measurement of Circulating Markers

Patient serum samples were collected after fasting overnight just prior to the breast core biopsy (and for the post-metformin sample 2 hours post-dose). Fasting glucose, insulin, c-peptide, c-reactive protein, leptin and adiponectin were measured using NHS biochemistry services to standardised and validated protocols. The homeostasis model assessment (HOMA) was calculated using the following equation: (glucose mmol/L ^∗^ insulin mU/L)/22.5.

IL-6 and TNF-alpha were measured in duplicate by High Sensitivity enzyme linked immunosorbent assay (ELISA) (Invitrogen). These assays utilise two amplification steps, allowing for the detection of low levels of cytokines present in serum and plasma samples.

#### Immunohistochemistry

Staining for p-AMPK (1 in 200, thr172 residue, Cell Signaling Technologies #2535) and pAKT (1 in 100, ser473 residue, Cell Signaling Technologies #4060), was performed on a Leica Bond-max autostainer in the GCP laboratory, Department of Pharmacology, University of Oxford. For pAMPK, cell pellet controls were generated using MCF7 cells treated with either 20 μM Compound C (negative control) or 250 μM AICAR (positive control) for 24 hours prior to harvesting. For pAKT, cell pellet controls were generated using serum starved MCF7 cells either untreated (negative control) or treated with IGF-1 (positive control) for 30 minutes prior to harvesting and the generation of a formalin fixed, paraffin embedded cell pellet block.

Quantitative scoring of the staining of complete tumour sections was evaluated by two accredited pathologists using high power fields the intensity of the immunostaining was classified into 4 categories: 0, no immunostaining present; 1, weak staining; 2, moderate staining; and 3, strong staining and the percentage of positive cells at each intensity was then classified into 4 groups; 1 (0-10% positive cells), 2 (11% to 50% positive cells), 3 (51% to 80% positive cells) or 4 (81 to 100% positive cells). The H-score of immunoreactivity was obtained by multiplying the intensity and percentage scores.

### Quantification and Statistical Analysis

Processing for dynamic PET-CT and gene expression profiling are reported above. Absolute difference in metabolites was analysed using paired non-parametric method (Wilcoxon Signed Rank Sum). All other differences in measurements pre- and post-metformin were compared using paired t-tests. Correlation analyses between gene expression scores, metabolites, metformin levels, circulating metabolic markers and K_FDG_ were performed using non-parametric methods (Spearman’s rank correlation coefficient). Differences in measurements between the OTR and FR groups were examined using an unpaired two-tailed t-test. Statistical tests for each analysis are defined in figure legends. In all cases a p-value <0.05 was considered significant. Analysis of RNASeq and Metabolomic data was carried out using non-parametric approaches and hence the data was not required to follow an underlying distribution. Kinetic model fits to the tumour TACs extracted from dynamic PET scans were runs-tested as a non-parametric check on fit quality. Significances of pre/post metformin differences in PET tracer kinetics parameters and normalised static uptake values were assessed using a parametric t-test and a non-parametric Wilcoxon signed rank test, the assumption of normally-distributed data underlying the t-test being rejectable with p<.05 for all variables except K1 according to the Shapiro-Wilk test. Statistical packages, GraphPad PRISM v6.0, R v3.3.1 and Matlab were used for analyses.

#### Sample Size Estimation

Based on our and others’ previous analyses of microarray data, a minimum of 20 cases with paired measurements at two time points were estimated to be sufficient to observe expression changes of at least 1.7-fold in genes showing a coefficient of variation at each time point up to 50% with a significance level after multiple test correction of p=0.05 (taking into account filtering of not expressed transcripts) and an 80% power. This estimate assumed uniformity of drug response. However, double the number was desirable for higher significance and considering correlation with baseline expression and response.

### Data and Software Availability

The RNASeq gene expression data reported in this paper have been reported in Mendeley data with address http://dx.doi.org/10.17632/cytrpb62f2.1.

### Additional Resources

ClinicalTrials.gov Identifier: NCT01266486.

## Supplemental Information

Document S1. Figures S1–S5 and Tables S1–S6

Document S2. Article plus Supplemental Information
