# Supplementary material for: Integrated Pharmacodynamic Analysis Identifies Two Metabolic Adaption Pathways to Metformin in Breast Cancer
Source: Cell Metab. 2018 Nov 6;28(5):679–688.e4. doi: 10.1016/j.cmet.2018.08.021 (PMC6224605; doi:10.1016/j.cmet.2018.08.021)
Supplement: Document S1. Figures S1–S5 and Tables S1–S6 [file mmc1.pdf]

Cell Metabolism, Volume *28*

Supplemental Information

Integrated Pharmacodynamic Analysis Identifies Two Metabolic Adaption Pathways to Metformin in Breast Cancer

Simon R. Lord, Wei-Chen Cheng, Dan Liu, Edoardo Gaude, Syed Haider, Tom Metcalf, Neel Patel, Eugene J. Teoh, Fergus Gleeson, Kevin Bradley, Simon Wigfield, Christos Zois, Daniel R. McGowan, Mei-Lin Ah-See, Alastair M. Thompson, Anand Sharma, Luc Bidaut, Michael Pollak, Pankaj G. Roy, Fredrik Karpe, Tim James, Ruth English, Rosie F. Adams, Leticia Campo, Lisa Ayers, Cameron Snell, Ioannis Roxanis, Christian Frezza, John D. Fenwick, Francesca M. Buffa, and Adrian L. Harris

Supplemental information

Integrated pharmacodynamic analysis identifies two metabolic adaption pathways to metformin in breast cancer

Authors:

Simon. R. Lord, Wei-Chen. Cheng, Dan. Liu, Edoardo. Gaude, Syed. Haider, Tom. Metcalf, Neel. Patel, Eugene. J. Teoh, Fergus. Gleeson, Kevin. Bradley, Simon. Wigfield, Christos. Zois, Daniel. R. McGowan, Mei-Lin. Ah-See, Alastair. M. Thompson, Anand. Sharma, Luc. Bidaut, Michael. Pollak, Pankaj. G. Roy, Fredrik. Karpe, Tim. James, Ruth. English, Rosie. F. Adams, Leticia Campo, Lisa. Ayers, Cameron Snell, Ioannis Roxanis, Christian. Frezza, John. D. Fenwick, Francesca. M. Buffa, Adrian. L. Harris

Inventory of Supplemental Information

SUPPLEMENTAL FIGURES:

Figure S1.

Figure S2.

Figure S3.

Figure S4.

Figure S5.

SUPPLEMENTAL TABLES:

Table S1.

Table S2.

Table S3.

Table S4.

Table S5.

Table S6.

SUPPLEMENTAL FIGURES:

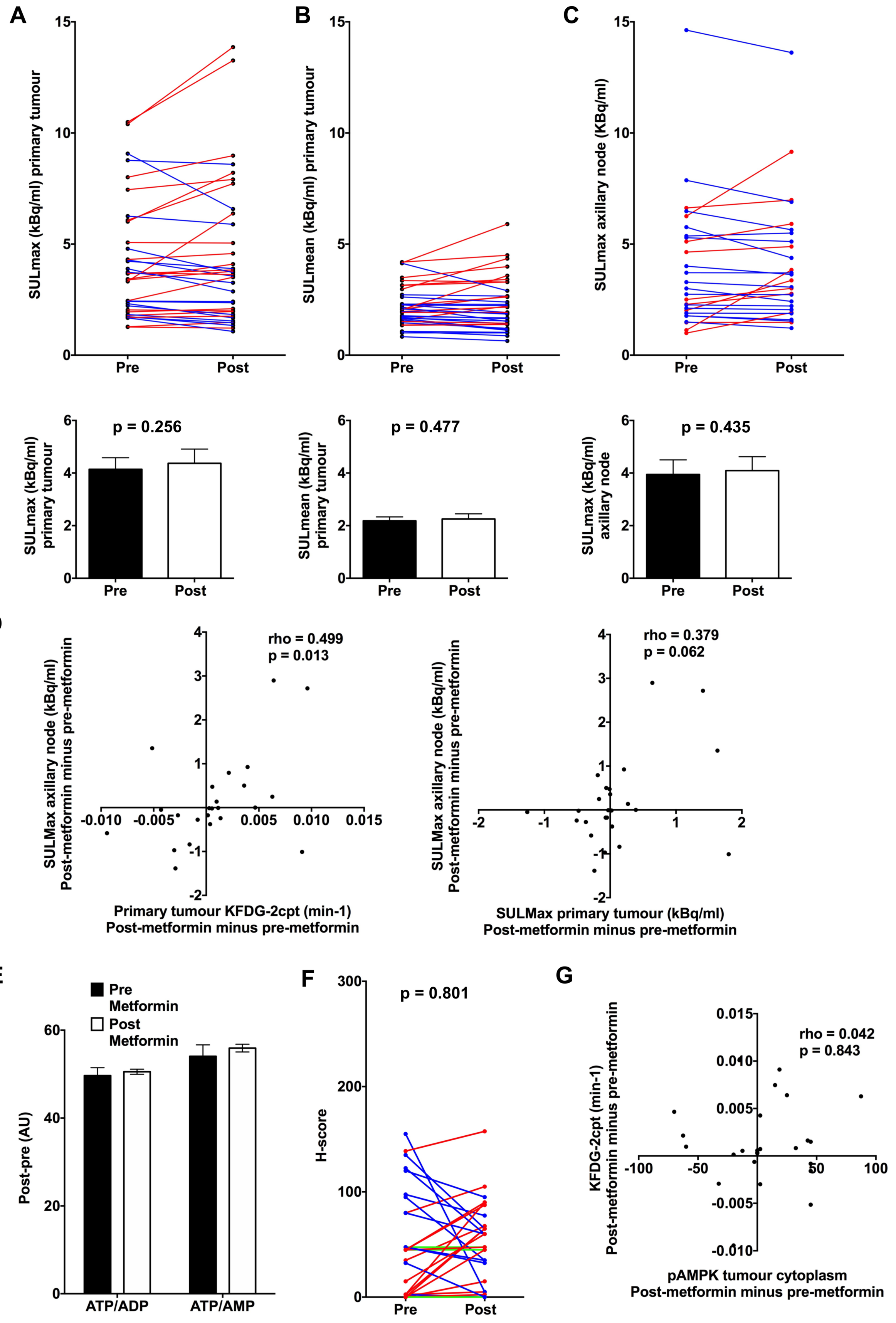

Fig. S1. Effect of metformin on standardised uptake value of primary breast tumour. Related to Figure 1.

Change in the (**A**) maximum and (**B**) mean standardised uptake value normalised to lean body mass of the primary tumour in individual patients (upper panel) and overall (lower panel; data shown are mean ± SEM) pre- and post-metformin (n=36). (**C**): Change in maximum standardised uptake value normalised to lean body mass of axillary nodes in individual patients pre- and post-metformin (n=27). (**D**): Correlation between change in $K_{FDG\text{-}2cpt}$ (post-metformin minus pre-metformin) and SUL_{Max} for the breast primary tumour, respectively, and change in SUL_{Max} for FDG avid axillary lymph nodes. Spearman's rank correlation coefficient and significance, are shown. (**E**): ATP/AMP and ATP/ADP ratios pre- and post-metformin (n=29); data shown are mean ± SEM. (**F**): Change in pAMPK of primary tumour measured by immunohistochemistry in individual patients pre- and post-metformin (n=32; red = increase, blue = decrease and green = no change). (**G**): Correlation between change in $K_{FDG\text{-}2cpt}$ and pAMPK for the breast primary tumour (both post-metformin minus pre-metformin). Spearman's rank correlation coefficient and significance, are shown.

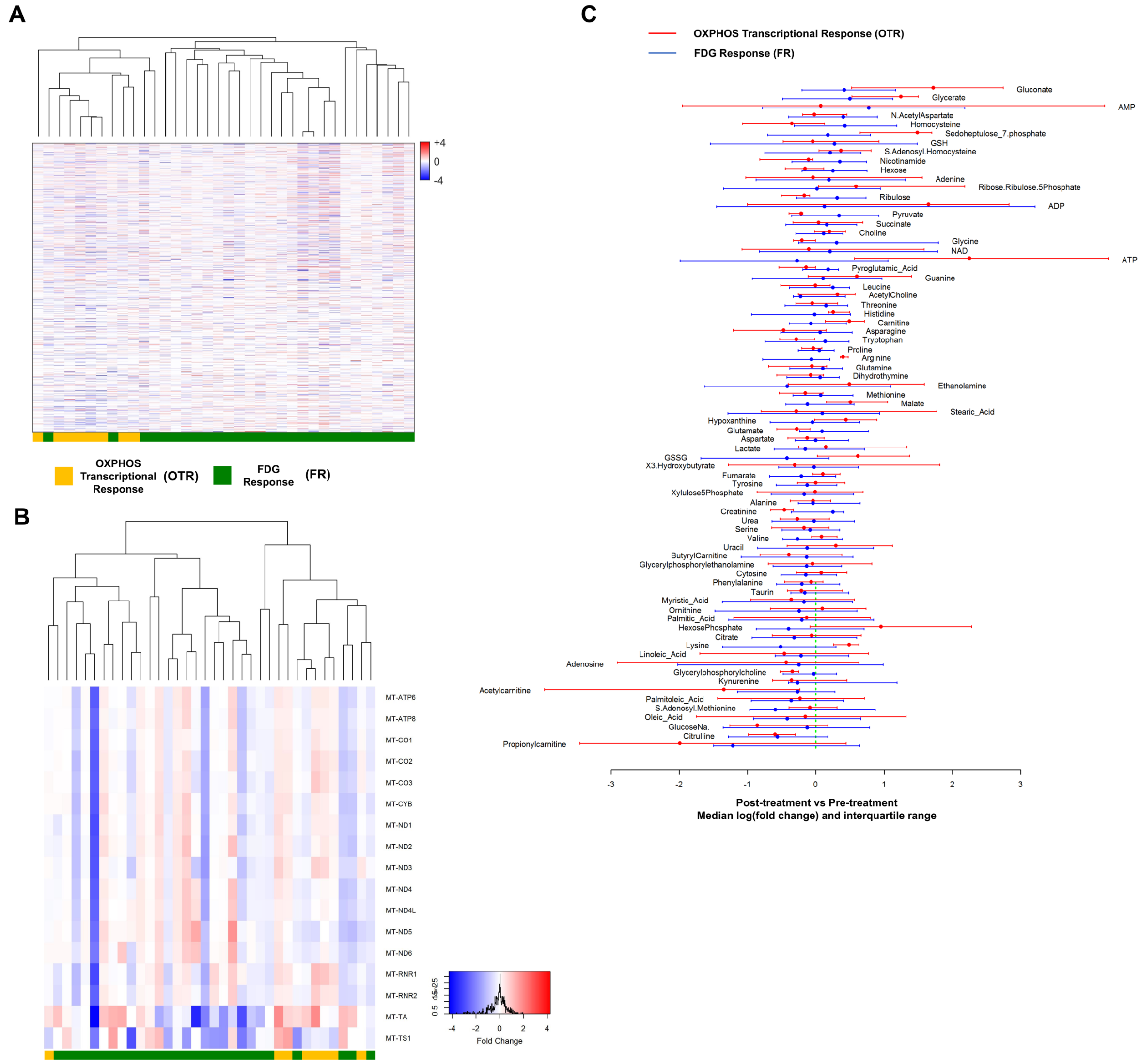

Fig. S2. Heatmaps of all expressed nuclear and mitochondrial encoded genes following metformin. Related to Figure 2.

Heatmaps of all expressed nuclear (**A**) and mitochondrial (**B**) encoded genes following metformin. Each row represents a gene and each column represents a single patient. Colours reflect the fold change for each gene post-metformin: Red = up-regulation, Blue = down-regulation. Samples were visually clustered using hierarchical clustering (n=36). Patients in OTR (orange) and FR (green) groups shown below. (**C**): Median log FC and interquartile range for metabolites in OXPHOS transcriptional response (OTR) and FDG response groups.

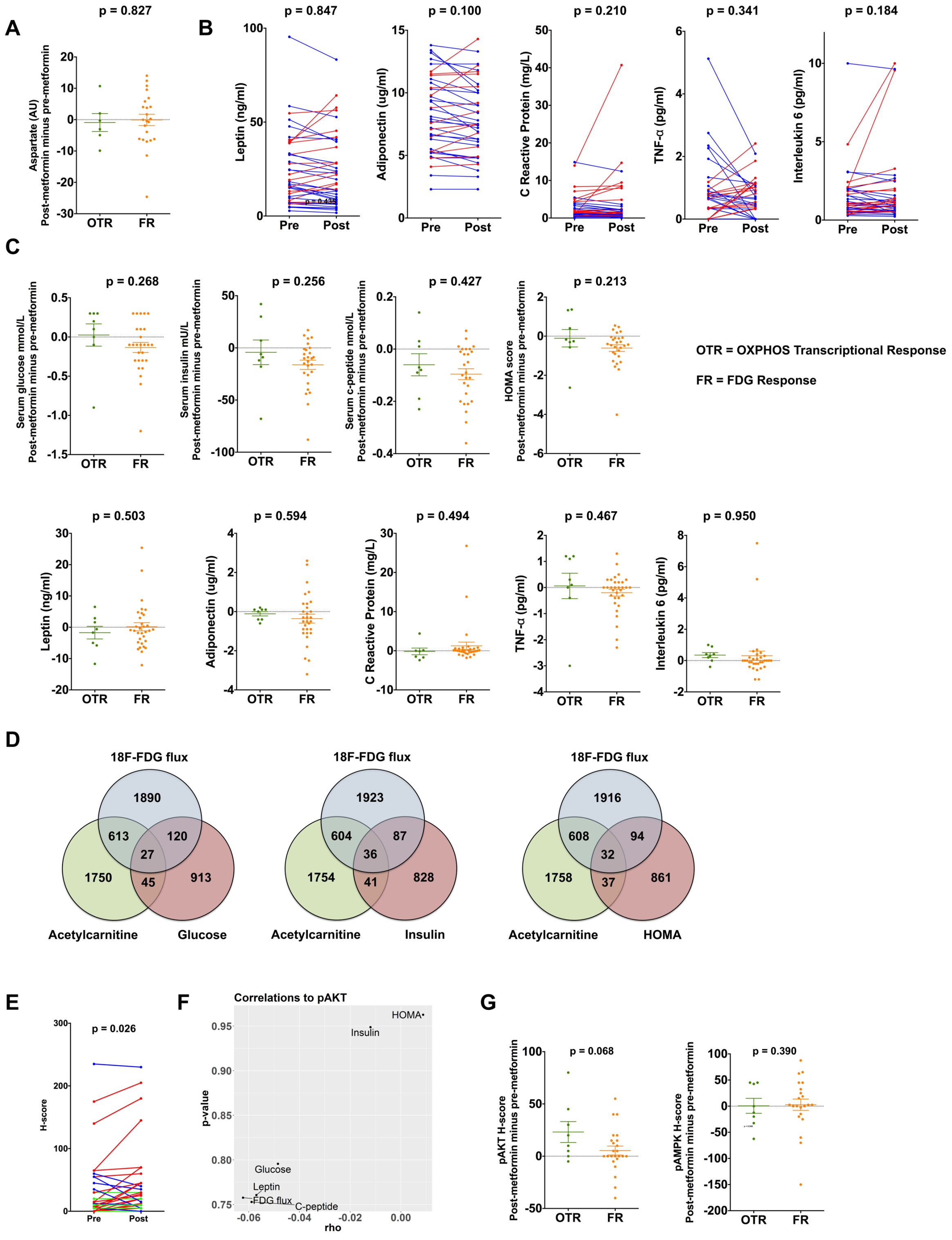
A
p = 0.827
Aspartate (AU) Post-metformin minus pre-metformin
OTR
FR
B
p = 0.847
Leptin (ng/ml)
p = 0.100
Adiponectin (ug/ml)
p = 0.210
C Reactive Protein (mg/L)
p = 0.341
TNF-α (pg/ml)
p = 0.184
Interleukin 6 (pg/ml)
Pre
Post
C
p = 0.268
Serum glucose mmol/L Post-metformin minus pre-metformin
p = 0.256
Serum insulin mU/L Post-metformin minus pre-metformin
p = 0.427
Serum c-peptide nmol/L Post-metformin minus pre-metformin
p = 0.213
HOMA score Post-metformin minus pre-metformin
OTR = OXPHOS Transcriptional Response
FR = FDG Response
p = 0.503
Leptin (ng/ml)
p = 0.594
Adiponectin (ug/ml)
p = 0.494
C Reactive Protein (mg/L)
p = 0.467
TNF-α (pg/ml)
p = 0.950
Interleukin 6 (pg/ml)
D
18F-FDG flux
1890
613
120
27
1750
45
913
Acetylcarnitine
Glucose
18F-FDG flux
1923
604
87
36
1754
41
828
Acetylcarnitine
Insulin
18F-FDG flux
1916
608
94
32
1758
37
861
Acetylcarnitine
HOMA
E
p = 0.026
H-score
Pre
Post
F
Correlations to pAKT
HOMA
Insulin
Glucose
Leptin
FDG flux
C-peptide
p-value
rho
G
p = 0.068
pAKT H-score Post-metformin minus pre-metformin
p = 0.390
pAMPK H-score Post-metformin minus pre-metformin
OTR
FR

Fig. S3. Relationship between systemic effects of metformin and tumour metabolic response. Related to Figure 3.

(**A**): Scatter plot to show change in tumour aspartate levels for the OTR and FR groups (post-metformin minus pre-metformin). Data shown are mean ± SEM and p-value on unpaired t-test (n=29). (**B**): Pre- and post-metformin levels of serum leptin, adiponectin, C-reactive protein, tumour necrosis factor-alpha and interleukin-6 for individual patients (n=40). (**C**): Scatter plots to show for the OTR and FR groups change in the systemic metabolic markers, serum glucose, serum insulin, serum c-peptide, HOMA, leptin and adiponectin, and the systemic inflammatory markers, C-reactive protein, tumour necrosis factor-alpha and interleukin-6 (all post-metformin minus pre-metformin). Data shown are mean ± SEM and p-value on unpaired t-test (n=36). (**D**): Venn diagram to show overlap of all genes whose change in expression correlated with change in tumour $K_{FDG\text{-}2cpt}$ and tumour acetylcarnitine and either HOMA, or systemic levels of circulating glucose or insulin. (**E**): Change in pAKT of primary tumour measured by immunohistochemistry in individual patients pre- and post-metformin (n=32; red = increase, blue = decrease and green = no change). (**F**): Relationship between change in tumour pAKT and circulating metabolic markers (all post-metformin minus pre-metformin). Spearman's rank correlation coefficient and significance, are shown (n=27). (**G**): Scatter plots to show change in pAKT and pAMPK of primary tumour for the OTR and FR groups (both post-metformin minus pre-metformin). Data shown are mean ± SEM and p-value on unpaired t-test (n=32). (**H**): Examples of pAKT immunohistochemical staining for individual patients, upper panel: increase after metformin; lower panel: no change after metformin.

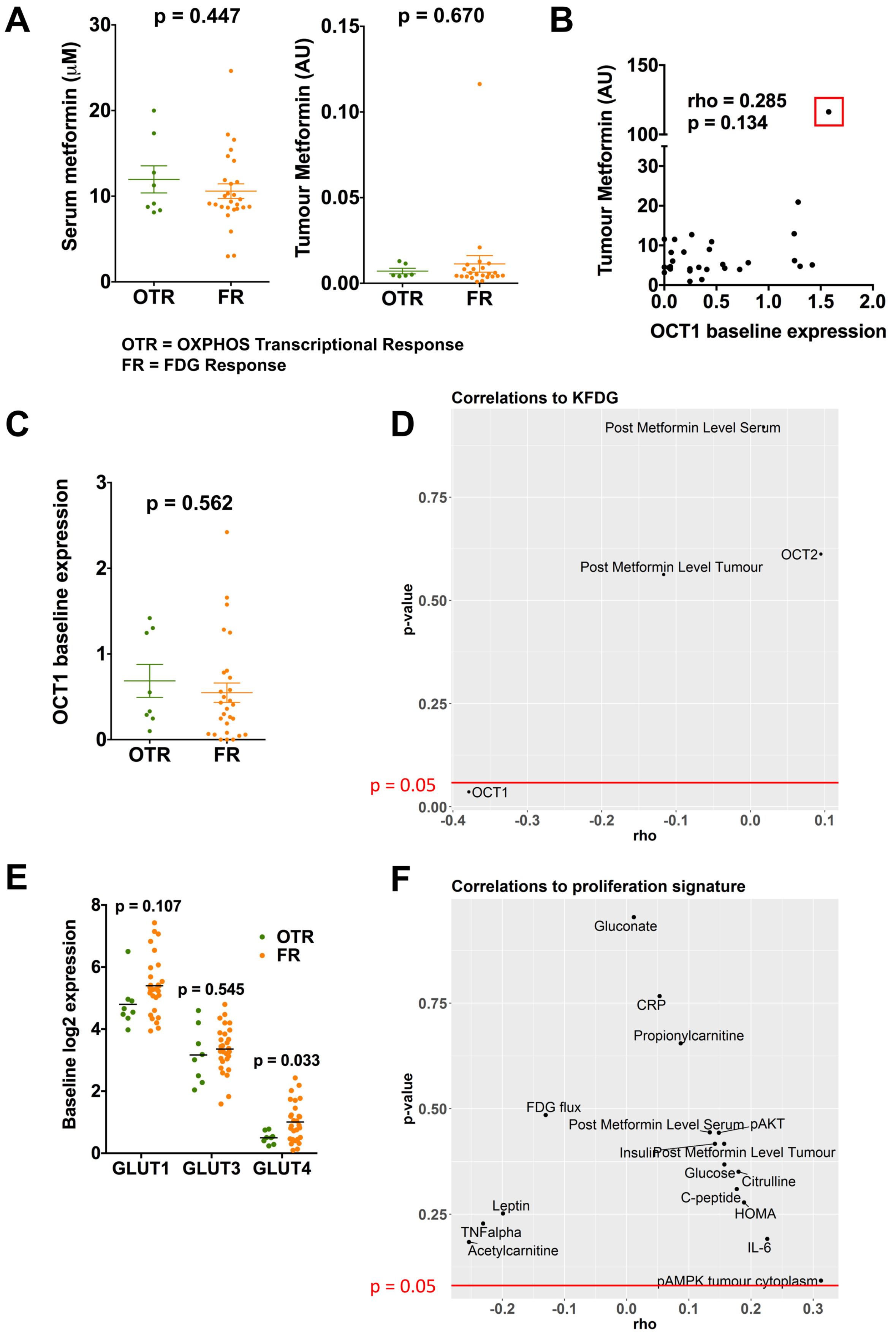
A
p = 0.447
Serum metformin (μM)
30
20
10
0
OTR
FR
p = 0.670
Tumour Metformin (AU)
0.15
0.10
0.05
0.00
OTR
FR
OTR = OXPHOS Transcriptional Response
FR = FDG Response
B
rho = 0.285
p = 0.134
Tumour Metformin (AU)
150
100
30
20
10
0
0.0
0.5
1.0
1.5
2.0
OCT1 baseline expression
C
p = 0.562
OCT1 baseline expression
3
2
1
0
OTR
FR
D
Correlations to KFDG
Post Metformin Level Serum
OCT2
Post Metformin Level Tumour
OCT1
p = 0.05
p-value
0.75
0.50
0.25
0.00
-0.4
-0.3
-0.2
-0.1
0.0
0.1
rho
E
p = 0.107
p = 0.545
p = 0.033
OTR
FR
Baseline log2 expression
8
6
4
2
0
GLUT1
GLUT3
GLUT4
F
Correlations to proliferation signature
Gluconate
CRP
Propionylcarnitine
FDG flux
Post Metformin Level Serum
pAKT
Insulin
Post Metformin Level Tumour
Glucose
Citrulline
C-peptide
HOMA
Leptin
TNFalpha
Acetylcarnitine
IL-6
pAMPK tumour cytoplasm
p = 0.05
p-value
0.75
0.50
0.25
-0.2
-0.1
0.0
0.1
0.2
0.3
rho

Fig. S4. Relationship between systemic effects of metformin and tumour metabolic/proliferation response. Related to Figure 3.

(**A**): Scatter plots to show serum (n=35) and tumour metformin levels (n=29) for the OTR and FR groups. Data shown are mean ± SEM, on unpaired t-test. (**B**): Correlation between tumour metformin levels and OCT1 baseline expression, patient with highest OCT1 expression and metformin level indicated Venn diagram to show overlap of all genes whose change in expression correlated with change in tumour $K_{FDG\text{-}2cpt}$ and tumour acetylcarnitine and either HOMA, or systemic levels of circulating glucose or insulin. (**C**): Scatter plot to show baseline OCT1 gene expression for the OTR and FR groups. Data shown are mean ± SEM, on unpaired t-test. (n=36) (**D**): Relationship between change in tumour $K_{FDG\text{-}2cpt}$ and OCT1, OCT2, tumour and serum metformin levels (all post-metformin minus pre-metformin). Spearman's rank correlation coefficient and significance, are shown. (**E**): Scatter plot to show for the OXPHOS transcriptional response group (OTR) and FDG response group (FR) change in GLUT1, GLUT3, and GLUT4 expression (log2FC) for the breast primary tumour (GLUT2 not expressed in most tumours). Data shown are mean ± SEM, unpaired t-test (n=36). (**F**): Relationship between change in proliferation gene signature (log2FC) with circulating or tumour immunohistochemical markers, metformin levels, K_{FDG}, or significantly altered tumour metabolites. Spearman's rank correlation coefficient and significance, are shown.

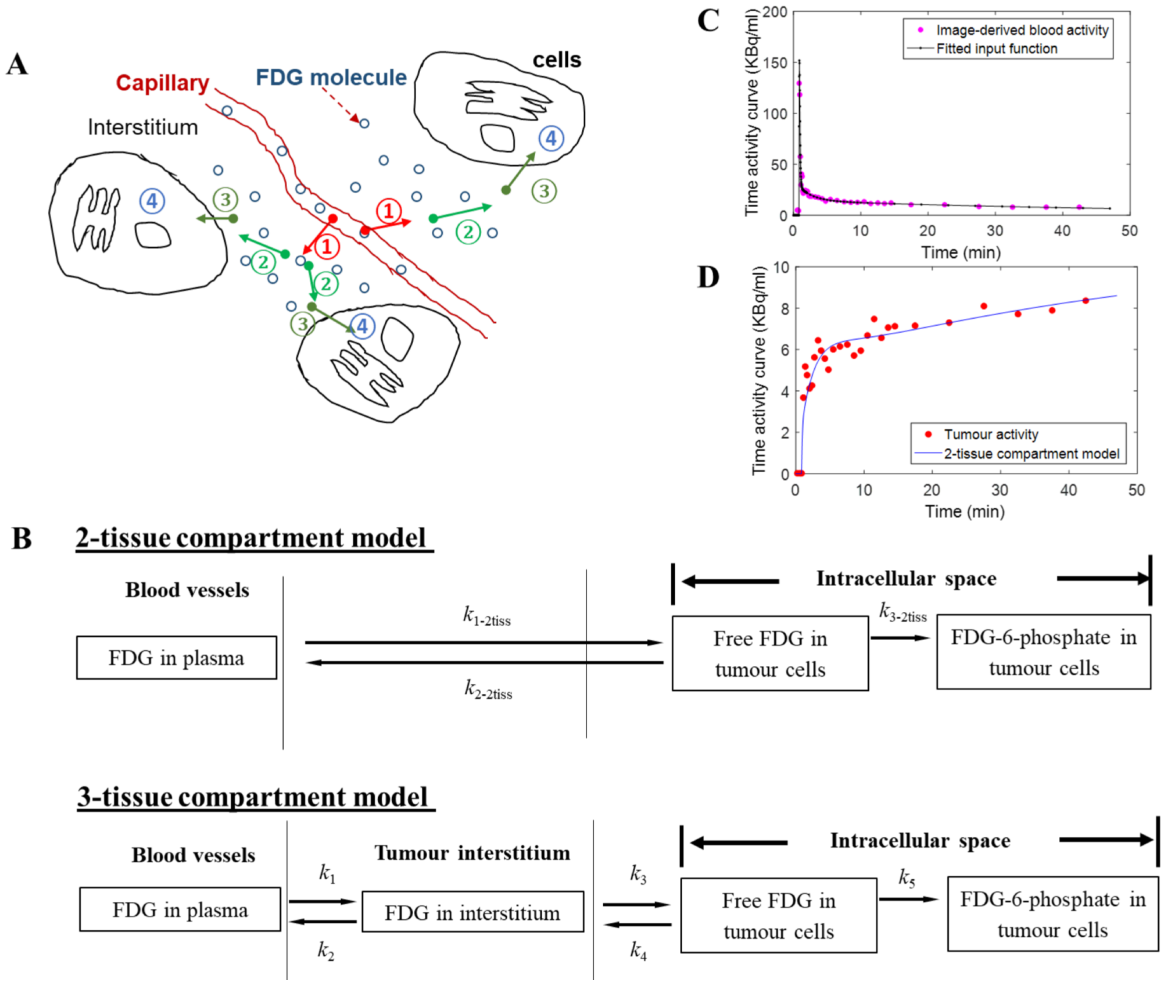

Fig. S5. Mechanism and modelling of 18F-FDG. Related to 'Dynamic PET-CT analysis' of STAR methods section.

(**A**) 18F-FDG tumour uptake occurs via: ① trans-capillary exchange; ② diffusion through the tumour interstitium; ③ trans-membrane transport to tumour intracellular spaces; and ④ intracellular phosphorylation of 18F-FDG. (**B**): two- and three-tissue compartment models describing 18F-FDG tumour uptake. (**C**): example of an image-derived blood input function, and (**D**): fit of the irreversible 2-tissue compartment model (continuous curve) to FDG uptake time-course data (dots) extracted from dynamic images for one patient.

SUPPLEMENTAL TABLES:

Inclusion criteria	Exclusion criteria
Women with a histology proven in situ primary breast cancer ≥2 cm in diameter	Radiotherapy, major surgery, significant traumatic injury, endocrine therapy, immunotherapy, chemotherapy or experimental therapy during four weeks prior to starting or during trial
Eastern Cooperative Oncology Group (ECOG) performance status 0–1	Pregnancy or breast feeding
Age ≥18 years	History of type 1 or type 2 diabetes
Fasting or random serum glucose less than 7.0 mmol/L	Treatment with metformin in the past year
No prior treatment for breast cancer and scheduled to commence neoadjuvant chemotherapy in ≤3 weeks time	Estimated glomerular filtration rate <45ml/min
Have given written informed consent and are capable of cooperating with protocol	Acute or chronic metabolic acidosis
Adequate bone marrow, renal and liver function	Known hypersensitivity to metformin

Table S1. List of key inclusion and exclusion criteria. Related to Figure 1.

			Number of patients
**Recruitment and samples analysed**	Total patient recruitment to study		41
	Number of paired PET-CT scans available for analysis		36
	Number of paired tumour samples with sufficient material for analysis	Metabolomics	29
		RNASeq	36
**ER/HER2 status**	ER positive		32
	ER negative		9
	HER2 positive		8
	HER2 negative		33
	Triple negative (ER negative and HER2 negative)		8
**Tumour type**	Ductal carcinoma		32
	Lobular carcinoma		7
	Mixed ductal and lobular carcinoma		2
	Grade 1		2
	Grade 2		24
	Grade 3		15
	Median tumour size (on magnetic resonance imaging)		49mm (range 30–147)
**Patient characteristics**	Median age at study entry		49 years (range 27–67)
	Median body mass index		28.1 (range 19.6–45.3

Table S2. Tumour and patient characteristics (for the 29 paired samples included in the general metabolomics analysis). Related to Figure 1.

Dynamic Imaging Variable	p-value		
	**Paired t-test**	**Wilcoxon**	**Mann-Whitney**
K1	0.145	0.162	0.341
k2	0.055	0.128	0.521
k3	0.343	0.053	0.392
Kflux (KFDG-2cpt(min-1)	0.041	0.027	0.510
SUVmean	0.918	0.271	0.356
TBRmean	0.255	0.540	0.540
MRglu	0.141	0.285	0.285

Table S3. P-values for all dynamic imaging variables using 3 different statistical tests, 2-tailed paired t-test; 2-sided Wilcoxon signed rank test; Mann-Whitney U-test. Related to Figure 1.

Pathway	KEGG ID	p-value*
Peroxisome	04146	<0.001
Arginine & proline metabolism	00330	<0.001
Valine, leucine & isoleucine degradation	00280	<0.001
Pyruvate metabolism	00620	0.001
Glutathione metabolism	00480	0.002
Citrate cycle	00020	0.004
Propanoate metabolism	00640	0.005
Fatty acid degradation	00071	0.005
Alanine & aspartate & glutamate metabolism	00250	0.005
Cysteine & methionine metabolism	00270	0.007
Lysine degradation	00310	0.009
Glycine, serine & threonine metabolism	00260	0.011
Huntingdon's disease	05016	0.016
Histidine metabolism	00340	0.023
PPAR signalling pathway	00320	0.030
Oxidative phosphorylation	00190	0.033
Ascorbate & aldarate metabolism	00053	0.033
Alzheimer's disease	05010	0.034
Glycolysis & gluconeogenesis	00010	0.040

Table S4. List of KEGG pathways linked to mitochondrial metabolism that were significantly upregulated following metformin treatment. * corrected Hypergeometric p-value. Related to Figure 2.

Circulating marker	Pre-metformin		Post-metformin		p-value*
	**Mean**	**SEM**	**Mean**	**SEM**	
Glucose (mmol/L)	4.94	0.08	4.82	0.45	0.032
Insulin (mU/L)	81.0	8.01	70.2	6.93	0.005
C-peptide (nmol/L)	0.59	0.04	0.50	0.03	<0.001
HOMA score	2.60	0.28	2.17	0.22	0.006
Leptin (ng/ml)	24.3	3.05	24.1	3.08	0.847
Adiponectin (ug/ml)	8.26	0.49	7.92	0.48	0.100
C-reactive protein (mg/L)	2.75	0.54	3.76	1.12	0.210
Tumour necrosis factor alpha (pg/ml)	0.74	0.16	0.60	0.10	0.341
Interleukin 6 (pg/ml)	1.50	0.26	1.82	0.40	0.184

Table S5. List of circulating markers tested. SEM, standard error of mean. * 2-tailed paired t-test. Related to Figure 3.

Gene	Full name	Brite hierarchy	
COX7B	cytochrome c oxidase subunit 7B	Energy metabolism	Oxidative phosphorylation
NDUFA4	NDUFA4, mitochondrial complex associated	Energy metabolism	Oxidative phosphorylation
NDUFS4	NADH:ubiquinone oxidoreductase subunit S4	Energy metabolism	Oxidative phosphorylation
AMY2B	amylase, alpha 2B (pancreatic)	Carbohydrate metabolism	Starch and sucrose metabolism
FBP1	fructose-bisphosphatase 1	Carbohydrate metabolism	Glycolysis / Gluconeogenesis
			Pentose phosphate pathway
			Fructose and mannose metabolism
GALK1	galactokinase 1	Carbohydrate metabolism	Galactose metabolism
			Amino sugar and nucleotide sugar metabolism
GYS1	glycogen synthase 1	Carbohydrate metabolism	Starch and sucrose metabolism
MGAM2	maltase-glucoamylase 2 (putative)	Carbohydrate metabolism	Galactose metabolism
			Starch and sucrose metabolism
PLCG1	phospholipase C gamma 1	Carbohydrate metabolism	Inositol phosphate metabolism
DNMT3B	DNA methyltransferase 3 beta	Amino acid metabolism	Cysteine and methionine metabolism
GCLC	glutamate-cysteine ligase catalytic subunit	Amino acid metabolism	Cysteine and methionine metabolism
			Glutathione metabolism
POLR2J2			Purine metabolism

	RNA polymerase II subunit J2	Nucleotide metabolism	Pyrimidine metabolism
POLR3GL	RNA polymerase III subunit G like	Nucleotide metabolism	Purine metabolism
			Pyrimidine metabolism
UBP1	beta-ureidopropionase 1	Nucleotide metabolism	Pyrimidine metabolism
		Metabolism of other amino acids	beta-Alanine metabolism
		Metabolism of cofactors and vitamins	Pantothenate and CoA biosynthesis
		Xenobiotics biodegradation and metabolism	Drug metabolism - other enzymes
HGSNAT	heparan-alpha-glucosaminide N-acetyltransferase	Glycan biosynthesis and metabolism	Glycosaminoglycan degradation
MAN2A2	mannosidase alpha class 2A member 2	Glycan biosynthesis and metabolism	N-Glycan biosynthesis
ST3GAL1	ST3 beta-galactoside alpha-2,3-sialyltransferase 1	Glycan biosynthesis and metabolism	Mucin type O-glycan biosynthesis
			Glycosaminoglycan biosynthesis - keratan sulfate
			Glycosphingolipid biosynthesis - globo and isoglobo series
			Glycosphingolipid biosynthesis - ganglio series
NADSYN1	NAD synthetase 1	Metabolism of cofactors and vitamins	Nicotinate and nicotinamide metabolism

Table S6. List of all KEGG annotated metabolism genes whose change in expression correlated with both change in tumour $K_{FDG\text{-}2cpt}$ and tumour acetylcarnitine levels. Related to Figure 3.
